# Improvement of poststroke cognitive impairment by intermittent theta bursts: A double‐blind randomized controlled trial

**DOI:** 10.1002/brb3.2569

**Published:** 2022-04-29

**Authors:** Wen Li, Qian Wen, Yu‐Han Xie, An‐Li Hu, Qing Wu, Yin‐Xu Wang

**Affiliations:** ^1^ Rehabilitation Medicine Department Affiliated Hospital of North Sichuan Medical College Sichuan China; ^2^ University of South China Hunan China; ^3^ Hubei University Of Economics WuHan China

**Keywords:** cognitive disorder, double‐blind randomized controlled trial, intermittent theta burst stimulation, stroke

## Abstract

**Background:**

Intermittent theta burst stimulation (iTBS) is known to improve cognitive impairment caused by Alzheimer's disease and Parkinson's disease, but studies are lacking with respect to the efficacy of iTBS on poststroke cognitive impairment (PSCI).

**Objective:**

This study was conducted to investigate the effect of left dorsolateral prefrontal cortex (DLPFC) iTBS on improving cognitive function in stroke patients.

**Methods:**

Fifty‐eight patients with PSCI are randomly divided into iTBS (*n* = 28) and sham stimulation groups (*n* = 30). Both groups receive routine cognitive‐related rehabilitation. The iTBS group is treated with iTBS intervention of the left DLPFC, and the sham stimulation group is treated with the same parameters at the same site for 2 weeks. Outcome measures are assessed at baseline (T0) and immediately after the last intervention (T1) by mini‐mental state examination (MMSE), Oxford cognitive screen, and event‐related potential P300.

**Results:**

There are no differences in baseline clinical characteristics between the two groups. After intervention, the MMSE scores and P300 amplitude increase significantly for both groups, and the P300 incubation period reduces significantly. The change value of the iTBS group is significantly higher than that of sham stimulation group (*p *< .05). Compared with the sham stimulation group, the iTBS group has more significant changes in semantic comprehension and executive function (*p* < .05).

**Conclusion:**

iTBS can effectively and safely improve overall cognitive impairment in stroke patients, including semantic understanding and executive function, and it also has a positive impact on memory function. Future randomized controlled studies with large samples and long‐term follow‐up should be conducted to further validate the results of the present study

## Graphical abstract

Study protocol and patient response to transcranial magnetic stimulation. (A) The stimulation target is shown, located on the patient's left dorsolateral prefrontal lobe. (B) Patients' scores of imitation, semantics and execution improved after the intervention. Imitation: patient magnetic patient gestures in minor mode. Semantics: Show the picture pointing to the specified object. Execution: Following the instruction to connect the graphics.

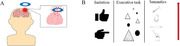

## 1 INTRODUCTION

Poststroke cognitive impairment (PSCI) is a common complication of stroke. Although some patients can recover cognitive functioning to varying degrees after stroke onset, roughly 24%−75% of stroke survivors have persistent cognitive impairment, which can worsen to poststroke dementia (Aben et al., 2018; K. Wang et al., 2021). PSCI patients suffer from the dysfunction of attention, executive function, memory, thinking, and language. This negatively affects their rehabilitation, prolongs the rehabilitation cycle, increases disability rates, and medical costs, and negatively affects their ability to live independently as well as their quality of life (Mchutchison et al., 2020; Rohde et al., 2019). At present, PSCI mainly focuses on drug therapy, for example, cholinesterase inhibitors (such as donepezil) and N‐methyl‐d‐aspartic acid receptor antagonists (such as carpalatin) (Gorelick et al., 2011; Kim et al., 2020; K. Wang et al., 2021). These drugs are effective in improving cognitive functioning but are often accompanied by adverse reactions (Farooq et al., 2017; Sun, 2018). The meta‐analysis results of nondrug therapy, such as cognitive training and psychological intervention, are not clear (Elliott & Parente, 2014; Merriman et al., 2019).

Repetitive transcranial magnetic stimulation (rTMS), as a noninvasive brain stimulation technique, modulates cortical excitability and synaptic structure and function to promote functional recovery in stroke patients (Rossini et al., 2015). In recent years, rTMS has been widely used to treat neuropsychiatric diseases, such as epilepsy, pain, depression, and Parkinson's disease. Studies have shown that high‐frequency rTMS applied to the left dorsolateral prefrontal cortex (DLPFC) can effectively and safely improve cognition impairment by inducing changes in neural activity in those suffering from Alzheimer's disease (X. Wang et al., 2020) and stroke (Waldowski et al., 2009; Yin et al., 2020) as well as improving the cognition in healthy individuals (Patel et al., 2020).

Intermittent theta burst stimulation (iTBS) is an optimization mode of rTMS but with a number of advantages, for example, low stimulus intensity, short stimulation cycle, and long‐term benefit (Nowak et al., 2010). Previous studies have shown that iTBS patterns improve overall cognitive function in healthy volunteers (Hoy et al., 2016; Wu et al., 2021) and in patients with Parkinson's disease (Trung et al., 2019). The cognitive function improvement can be attributed to a number of factors. On the one hand, iTBS reduces the inhibitory control of cone cells to increase the excitatory output and induces/enhances the neuroplasticity and excitability of the brain. On the other hand, cognitive function improvement is mediated by the association of DLPFC with the caudate nucleus and the stimulation‐induced increase in the neurotransmitter release (Hoy et al., 2016; Trung et al., 2019; Wu et al., 2021). iTBS is also gradually applied to PSCI. Szaflarski et al. included eight patients with chronic aphasia after stroke. After iTBS treatment in the left Broca area, the overall language ability of the patients improved (Szaflarski et al., 2011). A case report by Vuksanović et al. also showed positive results in iTBS application in an aphasia patient after stroke (Vuksanović et al., 2015). Therefore, iTBS seems to have a positive improvement effect on PSCI. However, the sample size of these studies is too small, and the cognitive scope of range is too single. Therefore, further research is needed to explore the effectiveness of iTBS on PSCI. In this study, a randomized double‐blind single‐center pseudo‐controlled trial was conducted to investigate the short‐term effects of iTBS on patients with PSCI.

## 2 METHODS

### 2.1 Subjects

In this study, patients with PSCI admitted to rehabilitation at the medicine department of the Affiliated Hospital of North Sichuan Medical College from January 10, 2020 to April 30, 2021 were recruited. Inclusion criteria consisted of the following: (1) patients who meet diagnostic criteria outlined in the *Diagnostic points of various cerebrovascular diseases* revised by the fourth National Cerebrovascular Disease Academic Conference of the Chinese Medical Association, confirmed by computed tomography (CT) or magnetic resonance imaging (MRI) and diagnosed as ischemic or hemorrhagic stroke by certified experienced doctors; (2) 18−65 years old with stroke duration between two and three months; (3) a PSCI diagnosis consistent with the diagnostic criteria of expert Consensus on the Management of PSCI (2017)—mini‐mental state examination (MMSE) score: illiteracy ≤17 score, primary school education ≤20 score; junior high school and above education ≤24 score; (4) no serious visual or hearing impairment and can complete relevant assessment and testing; (5) donepezil was administered 2 weeks after stroke to improve cognitive function; (6) stable vital signs; and (7) signed informed consent of patients and their families for iTBS treatment. Exclusion criteria consisted of the following: (1) patients with cognitive dysfunction caused by craniocerebral trauma or neurological diseases other than stroke; (2) patients with aphasia, unstable arrhythmias, or other serious physical conditions; (3) patients with contraindications of magnetic stimulation, such as wearing a pacemaker or intracranial metal; (4) patients with a history of seizures; (5) patients in critical condition with fever, infection, or vital organ failure; (and 6) postoperative patients with left cerebral hemorrhage.

### 2.2 Experimental design

This prospective single‐center randomized double‐blind pseudo‐controlled trial was approved by the Ethics Committee of the Affiliated Hospital of North Sichuan Medical College (approval no. 2021ER066‐1). Fifty‐weight patients were randomly divided into the iTBS group (*n* = 28) and the sham stimulation group (*n* = 30). Each subject received 10 consecutive rounds of iTBS or pseudo‐stimulation of DLPFC over 2 weeks once a day from Monday to Friday. All subjects received the same conventional cognition‐related medication and rehabilitation training, including donepezil 10 mg orally before bedtime and rehabilitation training including: (1) directional force training: the use of calendar, prompt card so that patients repeatedly identify, exercise patients on time and character of directional force; (2) attention training: arousing attention through visual tracking and deleting letter games; (3) computing power training: Each patient repeatedly performs calculations from easy to difficult problems; (4) memory training: through repeated recitation, PQRST method and picture memory training, and urge patients to use auxiliary tools such as notepad to help daily memory; (5) executive training: simulate the selection of travel routes, shopping, and other scenarios in daily life once a day for 30 min five times a week.

**FIGURE 1:**
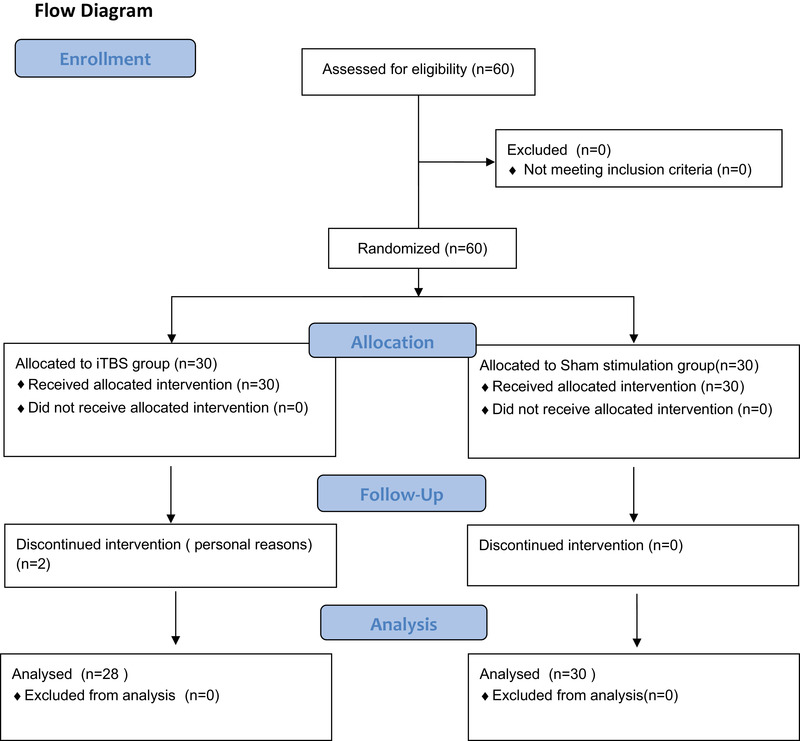
Participants’ flow diagram

All subjects were not aware of their grouping, and to minimize evaluator‐induced error, MMSE, Oxford cognitive screen (OCS), and P300 were assessed by the same professional therapist who was also unaware of grouping before treatment began. At the end of the last session, MMSE, OCS, and P300 were reassessed by the same professional therapist who did not know the grouping (Figure. 1).

## 3 INTERVENTION

### 3.1 Measurement of the resting motor threshold

Stimulation was performed using a transcranial magnetic stimulation system (nagneuro60 type stimulator, Nanjing Vishee Medical Technology Co., Nanjing, China) and a figure‐eight coil. The recording electrode was attached to the muscle abdomen of the abductor pollicis brevis on the normal side of the patient, and the reference electrode was placed at the tendon roughly 2 cm away from the recording electrode. The pole was placed proximal to the ipsilateral forearm. The coil was placed in the motor cortex of the healthy hemisphere with the coil tangent to the scalp and the patient sitting relaxed in a chair. The coils were shifted systematically in the primary motor cortex (M1) until a maximum and consistent motor‐evoked potential response was recorded from the abductor pollicis brevis on the normal side. The resting motor threshold (RMT) of the subjects was the minimum stimulus intensity that elicited at least a 50‐mV motor‐evoked potential in 5/10 consecutive cycles.

### 3.2 Stimulation plan

The left DLPFC (F3 point) was located according to the international 10/20 system, and the stimulus intensity was set to 100% RMT. iTBS parameters included three continuous pulses at 50 Hz repeated at 5 Hz (2 s on, 8 s off) for a total of 192 s and 600 pulses. In the sham stimulation group, the stimulation coil was rotated 90° perpendicular to the target area to generate minimum stimulation, and the stimulation parameters and sites were consistent with those of the iTBS group. Because the patients had not been treated with rTMS prior to this study, they did not know whether they received real or fake stimulation. Before disclosure at the end of subjects’ participation, they were asked whether they think they were in the iTBS or pseudo‐stimulation condition.

### 3.3 Outcome indicators

Cognitive function was assessed by MMSE, OCS, and event‐related potential (ERP) P300 before intervention (T0) and immediately after the last intervention (T1). The MMSE scale covers multiple cognitive areas. The MMSE measures orientation, memory, attention, computation, language, and visual ability (Ghafar et al., 2019; T. Zhang & Zhao, 2017). For MMSE, the higher the score, the better the cognitive function, and obtained scores ranged from 0 to 30. The following scores were considered as cognitive impairment: illiteracy score of ≤17, primary school education score of ≤20, and junior middle school or above score of ≤24. MMSE had good sensitivity (0.924) and specificity (0.806) in the assessment of cognitive impairment (Sleutjes et al., 2020). Considering the limitations of current assessment methods in assessing cognitive function in both patients with aphasia and spatial neglect, OCS has become a powerful tool to evaluate PSCI, including memory, language, number, practice, attention, and executive function, and it has high sensitivity and specificity (Blackburn et al., 2013).

ERP is the response of the brain to external stimuli. It reflects electrophysiological changes in the brain's attention and memory during the cognitive process, and, as such, it can be used as an effect index of iTBS to improve cognitive function (Pinto et al., 2018). P300, the most widely studied ERP component, mainly reflects processing speed and is therefore an important tool in studying memory related to physiological and pathological cognitive processes; its delay can be used as a marker of cognitive deterioration (Magnano et al., 2006).

All ERP tests were conducted in a dimly lit room with low sound and Evaluated by an experienced therapist. The subjects’ ears were stimulated at a sound pressure level of 85 dB, with a tone of 10 ms up or down, a plateau of 100 ms and 2.5 s apart. Auditory stimuli appeared randomly, and the tone of the target stimulus was set at 2000 Hz with a frequency of 20% (Han et al., 2018). The tone of the nontarget stimulus (standard stimulus) was set to 1000 Hz with an 80% frequency and a frequency of 0.5 Hz. The subjects were asked to distinguish between the two tones and to respond only to the target stimulus. Subjects were instructed to press a button on the joystick to respond to the target stimulus as quickly as possible (LynxONE Lynx Studio Technology, Inc.) (Gongora et al., 2021; Y. Zhang et al., 2021). Electroencephalography was recorded continuously with Ag/AgCl active electrode cap. According to the international 10/20 system, the electrode was placed at Cz point, the reference electrode was placed at A point on the right earlobe, the grounding electrode was placed at FPz point, and the impedance between the electrode and the skin was less than 5 KΩ. The raw data were analyzed using Butterworth zero‐phase filter, and the Bandpass filter was set between 1 and 30 Hz during analysis. We identified the presence of tasks (i.e., targets and criteria) as related stimuli based on stimulus initiation (−200 to 800 ms), after visual data, the semi‐automatic manual elimination of each section (gradient: 50 μV; maximum or minimum amplitude: −200 to 200 μV; minimum allowed movement interval: 0.5 μV). If the difference between the minimum and maximum amplitudes of a single segment exceeds 100 μV, the segment was tagged and deleted. If artifacts are detected, events were tagged 200 ms before and after the occurrence of accurate artifacts to control the noise source. In addition, independent component analysis was used to remove any blinks that are not encoded by the artifact algorithm (Stuckenschneider et al., 2020).

### 3.4 Sample size

In the pilot trial, 10 subjects were randomly assigned to receive iTBS or sham stimulation and were evaluated for MMSE before intervention (T0) and the last intervention (T1). At T1 on this basis, MMSE score was 18.67 ± 4.89 (mean ± SD) in iTBS group and 14.87 ± 4.04 (mean ± SD) in sham stimulation group. The sample size of 23 participants in each group was 0.8, which required testing for differences between assumed mean values at a significant level of 0.05. Given the short follow‐up time, it was assumed that 30% of patients in each group stopped treatment and/or lost follow‐up. Finally, the sample size of 30 people in each group was determined.

### 3.5 Randomization method

The iTBS and sham stimulation groups used a random number table to produce a randomly assigned sequence in a 1:1 ratio and generate sequentially numbered and opaque sealed envelopes. After baseline cognitive function assessment by evaluators, subjects were given a random identification number and assigned to treatment. In this study, subjects and evaluators were blinded. Outcome indicators were evaluated by rehabilitation therapists and physicians (nontrial recruiters and trial executors) who have received standardized training and professional technical qualification certificates.

### 3.6 Statistical analysis

All statistical analyses were performed using SPSS 22.0 (SPSS Inc., Chicago, IL, USA). The Shapiro–Wilk test was utilized to assess if the scores conformed a normal distribution. For quantitative data that conform to a normal distribution, the mean ± standard deviation ($\bar{x}$± s) was used. For quantitative data that did not conform to a normal distribution, interquartile range was used. The change value “Δ” was figured out to assess the differences of cognitive function scores along therapy timeline. For quantitative data conforming to normal distribution, one‐way analysis of variance (ANOVA) was used for comparison, for quantitative data not conforming to normal distribution, nonparametric test was used for comparison, and for count data, the chi‐square test was used for comparison. The Cohen's *d* and Morris ppc2 were used to assess effect size of all the variables. The significance level was set at 0.05.

## 4 RESULTS

### 4.1 Demographic and clinical characteristics

A total of 60 subjects were included. In the iTBS group, two subjects withdrew from the intervention due to private reasons unrelated to the study. Accordingly, 58 subjects completed the study. Table 1 lists the demographic and clinical information of the subjects. At baseline, there were no significant differences between the two groups in age, sex, stroke duration/type, the location of the lesion, hemiplegic limb, education level, MMSE, and P300 latency and amplitude (*p* > .05). At the end of the treatment, the subjects were asked if they knew they were in the iTBS or pseudo‐stimulation condition, and all said no. One subject in the iTBS group had sneezing symptoms during stimulation and the symptoms disappeared immediately after the stimulation was stopped, and no serious adverse events were recorded in remaining subjects.

**TABLE 1:** Baseline demographic and clinical characteristics

Characteristic	iTBS group (*n* = 28)	Sham stimulation group (*n* = 30)	*t*/*χ* ^2^/*Z* value	p Value
Age (years)	69.5 (60.0,78.0)	66.0 (53.0,75.0)	−0.935	.350
Sex (male/female)	16:12	18:12	0.049	.825
Poststroke duration (days)	25 (17,30)	25 (18,30)	−0.871	.851
Cortical/subcortical	14:14	12:18	0.586	.444
Hemorrhagic/ischemic stroke	10:18	16:14	1.818	.178
Side of paresis (left/right)	16:12	24:6	3.535	.06
Education level				
(Illiteracy/primary school/junior high school and above)	4:12:12	6:10:14	0.667	.716
MMSE	12.5 (8,19)	11 (7,15)	−0.843	.399
OCS				
Picture naming	2 (0,4)	2 (1,3)	−0.225	.822
Semantics	3 (2,4)	3 (3,4)	−0.650	.516
Orientation	2 (1,2)	2 (1,4)	−0.773	.439
Visual field	4 (2,4)	4 (4,4)	1.452	.146
Sentence reading	0 (0,4)	1 (0,4)	1.057	.290
Number writing and calculation	3 (2,5)	2 (1,4)	−1.351	.177
Imitation	8.5 (6,10)	7 (6,9)	−1.892	.059
Recall and recognition	3 (0,6)	3 (6,4)	0.032	.975
Executive task	2 (1,6)	2 (2,6)	0.478	.633
P300				
Latency (ms)	390.07 ± 22.40	389.67 ± 19.44	0.074	.942
Amplitude (μV)	5.25 (4.20,6.30)	5.50 (4.30,6.30)	0.749	.454

*Note*: Data were expressed as mean ± SD or median (interquartile range [IQR]).

Abbreviations: MMSE: mini‐mental state examination; iTBS, intermittent theta burst stimulation; OCS: Oxford cognitive screen; P300: P300 from event‐related potential measurements.

OCS is based on the previous five cognitive categories (memory, language, number, practice, attention and executive function), which are divided into picture naming, semantics, orientations, visual field, sentence reading, numbers, imitation gestures, recall, and execution modules. There were no significant differences between the two groups at baseline for MMSE, each module of OCS, and the latency and amplitude of P300. After treatment, MMSE scores (media difference = 17; 95% confidence interval [CI]: 1.00–6.00; effect size:0.644; *p *= .045) increased compared with baseline with statistically significant change (Table 2).

**TABLE 2:** Comparison of clinical outcomes between the two groups

Variables	Time	iTBS group (*n* = 28)	Sham stimulation group (*n* = 30)	Estimated difference	Lower 95% CI	Upper 90% CI	*Z* Value	p Value
MMSE								
	T0	12.5 (8,19)	11 (7,15)	1	−2	5	−0.843	.399
	T1	17 (14,24)	14 (11,18.75)	3	1	6	2.046	.045^*^
OCS								
Picture naming	T0	2 (0,4)	2 (1,3)	0	−1	1	−0.225	.822
	T1	3.5 (2,4)	3 (2,4)	0	0	0	0.167	.867
	△	0 (0,2)	1 (1,2)	−1	−1	0	1.573	.116
Semantics	T0	3 (2,4)	3 (3,4)	0	−1	0	0.650	.516
	T1	4 (4,4)	4 (3,4)	0	0	1	−2.198	.028^*^
	△	0 (0,2)	0 (0,1)	0	0	1	−1.816	.069
Orientation	T0	2 (1,3)	2 (1,4)	0	−1	0	0.773	.439
	T1	4 (3,4)	3 (3,4)	0	0	1	−1.118	.264
	△	1.5 (0,2)	1 (0,1)	0	0	1	−1.184	.236
Visual field	T0	4 (2,4)	4 (4,4)	0	0	0	1.452	.146
	T1	4 (3,4)	4 (4,4)	0	0	0	0.9639	.336
	△	0 (0,0)	0 (0,0)	0	0	0	−0.957	.339
Sentence reading	T0	0 (0,4)	1 (0,4)	0	−1	0	1.057	.290
	T1	2 (0,4)	2 (1,4)	0	−1	0	1.093	.274
	△	0 (0,1.75)	1 (0,1)	0	−1	0	0.667	.505
Number writing and calculation	T0	3 (2,4)	2 (1,4.25)	1	0	2	−1.351	.177
	T1	4.5 (3,6)	2 (4,4.25)	1	0	2	−1.486	.137
	△	2 (0,2)	1 (1.5,2)	0	−1	1	−0.388	.698
Imitation	T0	8 (6,10)	7 (6,9)	1	0	2	−1.892	.59
	T1	12 (10,12)	10 (9,11)	1	0	2	−2.745	.006^*^
	△	3 (2,4)	2 (2,3)	1	0	1	−1.262	.207
Recall and recognition	T0	3 (0,6)	3 (1,4)	0	−1	2	0.032	.975
	T1	5 (4,8)	5 (3,6)	0	−1	2	−0.568	.570
	△	2 (1,4)	2 (2,2)	0	−1	1	0.033	.874
Executive task	T0	2 (1,6)	2 (2,6)	0	−1	1	0.478	.633
	T1	6 (3,7)	3 (4,6)	1	0	3	−1.544	.123
	△	3 (1.25,4)	2 (1,2)	2	1	2	−2.921	.003^*^

*Note*: Data were expressed as median (interquartile range [IQR]). The asterisk indicates that a significant difference was observed in the data (*p < .05).

Abbreviations: CI, confidence interval; MMSE: mini‐mental state examination; OCS: Oxford cognitive screen.

For OCS, there were statistical differences in semantic (media difference = 4; 95% confidence interval [CI]: 0.00–1.00; effect size:0.497;*p *= .028) and imitation (media difference = 12; 95% confidence interval [CI]: 0.00–2.00; effect size:0.746; *p *= .006) between groups at T1. Due to the differences in some points test group between groups, we further conducted statistical analysis on the changes in scores of each module between groups. Results from the comparison of changes between groups suggest significant differences in executive function scores (media difference = 0; 95% CI: −1.00–0.00; effect size:0.811;*p *= .003), while imitative function (media difference = 0; 95% CI: 0.00–1.00; *p *= .069) and semantic (media difference = 3; 95% CI: 0.00–1.00; *p *= .207) scores become insignificant. For the other modules, the scores of each module were improved in both groups before and after intervention, but not significantly, and the difference was not statistically significant when comparing the change values between the two groups (*p* > .05) (Table 2).

For P300, there was a significant increase in the latency of P300 (mean difference = 345.71; 95% CI: −31.49–7.88; effect size: 0.947; *p *= .001) after intervention, but the amplitude (mean difference = 0.85; 95% CI: −0.03–1.72; effect size: 0.978; *p *= .057) showed no significant difference. Further statistical analysis of the changes in P300 between the two groups suggested that there were statistical differences in the changes both in the latency (media difference = −37.5; 95% CI: −14.9–9.00; effect size: 1.295; *p* < .001) and amplitude (media difference = 4.5; 95% CI: 0.50–1.90; effect size: 0.984; *p *= .001) of P300 as shown in Table 3.

**TABLE 3:** Event related potentials (ERPs)—P300 assessments

Variables	Time	iTBS group (*n* = 28)	Sham stimulation group (*n* = 30)	Estimated difference	Lower 95% CI	Upper 90% CI	*t*/*Z* Value	p Value	effect size
P300 latency (ms)									
	T0	390.07 ± 22.40	389.67 ± 19.44	0.40	−10.61	11.42	0.074	.942	
	T1	345.71 ± 25.12	365.40 ± 19.58	−19.69	−31.49	−7.88	−3.341	.001^*^	0.947
	△	−37.5 (−50,−30)	−24 (−30,−18)	−22	−14.5	−9	−3.91	<.001^*^	1.295
P300 amplitude (μV)									
	T0	5.25 (4.2,6.3)	5.50 (4.3,6.3)	−0.15	−0.8	0.3	0.749	.454	
	T1	9.45 (7.8,10.9)	9 (7.3,9.7)	0.8	0	1.7	−1.778	.075	0.479
	△	4.5 (3.4,5)	3.2 (2.5,3.9)	1.1	0.5	1.9	−3.370	.001^*^	0.984

*Note*: Data were expressed as mean ± SD or median (interquartile range [IQR]). The asterisk indicates that a significant difference was observed in the data (*p < .05).

Abbreviations: CI, confidence interval; P300: P300 from event‐related potential measurements.

## 5 DISCUSSION

This randomized controlled double‐blind trial provides initial evidence for the potential of iTBS to treat PSCI. In multiple assessments of neuroelectrophysiological and neuropsychological tests, iTBS stimulation of the left DLPFC improved overall cognitive function, with significant improvements in executive function. No significant differences were found between the two groups in serious adverse events and drop‐out rates. Mild cognitive impairment after stroke is a key therapeutic target as it can slow down the process of cognitive degeneration and prevent its development to vascular dementia (Gorelick et al., 2011). This study confirmed the regulation and recovery effect of iTBS with respect to cognitive function and further consolidated the potential efficacy of noninvasive brain stimulation technology on PSCI.

Synaptic plasticity is the most important biological mechanism leading to learning and memory, and long‐term potentiation (LTP) is a major neurophysiological factor related to learning and memory (Di Lorenzo et al., 2019). A key mechanism of cognitive dysfunction in Alzheimer's disease is LTP‐like cortical plasticity impairment, which is associated with cognitive decline (Di Lorenzo et al., 2020). Studies have shown that rTMS has a direct effect on the stimulation‐target region and can increase blood flow, promote the expression of neurotrophic factors (such as brain‐derived nerve‐growth factor and vascular endothelial nerve‐growth factor), and improve the release of neurotransmitters (such as acetylcholine, dopamine, norepinephrine, and serotonin) (Anderkova & Rektorova, 2014; Chou et al., 2020). Y. Li et al. (2020) used functional MRI (fMRI) to demonstrate that rTMS enhances functional connectivity between target and other areas in the cognitive‐processing network; it also enhances neural plasticity and neural‐activity changes. rTMS can also increase synaptic plasticity and regulate cortical excitability by inducing long‐term potentiation, which is related to γ‐aminobutyric acid‐mediated inhibition and the up‐regulation of N‐methyl‐d‐aspartic acid receptor activity (Chou et al., 2020; Gomes‐Osman et al., 2018). In addition, magnetic stimulation can promote the proliferation and neurogenesis of hippocampal cells in the dentate gyrus, which are related to memory and learning processes, thus improving cognitive function (Ueyama et al., 2011). The above neurophysiological changes have also been verified in studies related to the iTBS treatment of Parkinson's disease (Lang et al., 2020; Trung et al., 2019), Alzheimer's disease (Koch et al., 2014), and poststroke depression with cognitive impairment (Yi et al., 2020). Combined with the similar mechanism of iTBS and rTMS and the positive results of iTBS in cognitive impairment caused by other diseases, there are strong reasons to support the possible mechanism of iTBS. Studies on the mechanisms related to cognitive impairment suggest that changes in LTP‐like cortical plasticity are correlated with changes in cognitive function (X. Li et al., 2021). Therefore, in future PSCI studies, LTP evaluation can help to further explore the relevant mechanisms of iTBS to improve PSCI.

DLPFC, as a core region involved in executive functions such as working memory and cognitive flexibility, is a key node of the central executive network and is closely related to the regulation of executive functions (Baker et al., 2014). Tsai et al. have demonstrated that the overall cognitive and memory functions of patients with left hemisphere stroke can be enhanced after iTBS intervention (Tsai et al., 2020). And Pinto et al. have also reported that iTBS can improve working memory and executive function in healthy adults (Pinto et al., 2021), which is inconsistent with the results of the present study. The change of memory score before and after treatment in the two groups was higher in the iTBS group than in the sham stimulation group, but not significantly. This may be due to different sites and hemispheres, outcome indicators and the sample size of this study. Animal experiments have documented that θ‐wave oscillations are associated with working memory processes and play a key role in integrating the corresponding brain regions of working memory. Synchronous θ‐wave activity between the prefrontal and posterior parietal regions is associated with encoding working memory (Colgin, 2011; Sauseng et al., 2010). The specific θ‐wave rhythm stimulation released by iTBS may improve the coherence of neurons between the hippocampus and the prefrontal cortex, excite the corresponding cerebral cortex, and promote the induction of working memory processes (Noda et al., 2018, 2017). Therefore, randomized controlled studies with larger samples and more rigorous protocols are needed to further explore the corresponding conclusions, including mechanism exploration based on fMRI.

At present, there are few clinical applications of ERP as well as few ERP‐ and iTBS‐related studies. P300, as an electrophysiological indicator, was used in this study together with MMSE and OCS to assess cognitive functioning objectively and sensitively. P300 reflects the information processing of working memory and the speed processing involved in decision‐making. The incubation period is related to the information processing of external environment and reflects the speed of the brain's classification and recognition of external stimuli. The amplitude represents the excitement of the central nervous system during information recognition and processing (Lin et al., 2019; Rêgo et al., 2012). The results of this study show that, after iTBS intervention, the latency of P300 reduced, the amplitude of P300 increased, and the overall cognitive function of patients improved, which is consistent with previous research (Pinto et al., 2018). These improvements may be related to the iTBS‐mediated enhancement of neurotransmitter dopaminergic and glutamate connections (Anderkova & Rektorova, 2014).

This study has several limitations. First, iTBS is rarely used in the treatment of PSCI, and its parameters require further study, for example, stimulation frequency/duration and total number of pulses/courses. Second, in this study, a treatment time of 2 weeks was used, the patients were followed up after treatment, and the evaluation of long‐term iTBS efficacy on PSCI was lacking. In addition, there was no clear classification of lesion location and lesion size of patients in this cohort. Although there was no statistical difference in the comparison of baseline assessments, based on the lesion area and volume of individual differences, different types of strokes as well as the coil positioning problem may interfere with iTBS results, and iTBS function mechanism is not clear. Therefore, future studies should explore this to improve PSCI in combination with fMRI for accurate target localization. Finally, the sample size of this study is small, so a multi‐center randomized controlled study with a large sample is required to further explore the efficacy of iTBS in improving PSCI.

## 6 CONCLUSION

This randomized controlled trial provided evidence for the efficacy and tolerability of iTBS for the left DLPFC in the treatment of PSCI. After iTBS intervention, overall cognitive function improved, especially executive function, and there were improvements in memory function. Only one patient developed irritation‐related sneezing symptoms during treatment, and none of the remaining patients reported any adverse events. Based on these results, future studies with long‐term follow‐up are needed to further determine the role of iTBS in treating PSCI.

## CONFLICT OF INTEREST

The authors declare no conflict of interest.

### PEER REVIEW

The peer review history for this article is available at https://publons.com/publon/10.1002/brb3.2569.

## Data Availability

The data that support the findings of this study are available from the corresponding author upon reasonable request.
